# Fruitful Decades for Canthin-6-ones from 1952 to 2015: Biosynthesis, Chemistry, and Biological Activities

**DOI:** 10.3390/molecules21040493

**Published:** 2016-04-15

**Authors:** Jiangkun Dai, Na Li, Junru Wang, Uwe Schneider

**Affiliations:** 1 College of Science, Northwest A & F University, Yangling 712100, Shaanxi, China; daijkun@hotmail.com (J.D.); lnuk@nwsuaf.edu.cn (N.L.); 2 EaStCHEM School of Chemistry, The University of Edinburgh, The King’s Buildings, David Brewster Road, Edinburgh EH9 3FJ, UK

**Keywords:** canthin-6-one, biosynthesis, chemistry, biological activities

## Abstract

In this review, more than 60 natural canthin-6-one alkaloids and their structures are considered. The biosynthesis, efficient and classic synthetic approaches, and biological activities of canthin-6-one alkaloids, from 1952 to 2015, are discussed. From an analysis of their structural properties and an investigation of the literature, possible future trends for canthin-6-one alkaloids are proposed. The information reported will be helpful in future research on canthin-6-one alkaloids.

## 1. Introduction

**Figure 1:**
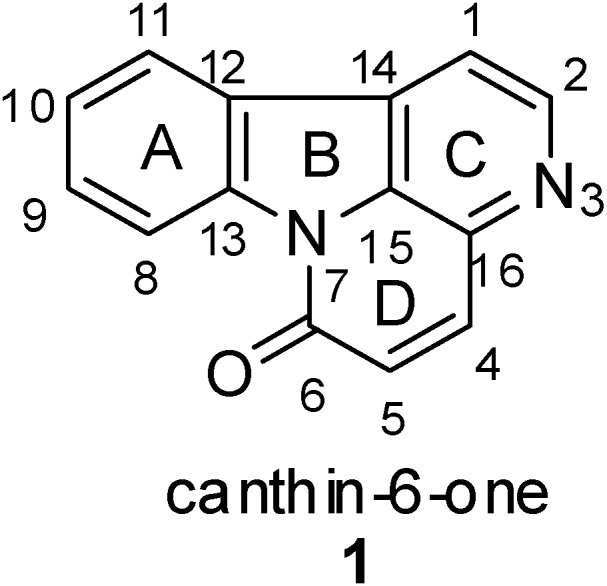
Structure of canthin-6-one **1**.

The canthin-6-one alkaloids, a subclass of β-carboline alkaloids with an additional D ring, have been isolated from various plants, principally those in the Rutaceae [1,2,3,4,5,6,7,8] and Simaroubaceae [9,10,11,12,13,14,15,16,17] families, but also those in the Amaranthaceae [18], Caryophyllaceae [19] and Zygophyllaceae [20] families, and more recently from fungi [21] and marine organisms [22]. Canthin-6-one (**1**) (Figure 1) was first isolated in 1952 by Haynes *et al.* [23] from the Australian tree *Pentaceras australis*. A literature search revealed that since then more than 60 members of this class of alkaloids have been isolated from natural sources (Table 1).

In 1986, Guicciardi’s group was the first to demonstrate the biosynthesis of canthinone-type alkaloids through feeding experiments in which ^14^C-tryptophan was administered to cell cultures of *Ailanthus altissima* [24], and this was confirmed by a study by Aragozzini’s group a few years later [25]. Based on the biosynthetic line of canthin-6-one alkaloids, the infractine-functionalized and nanoparticle-supported biomimetic synthesis of canthin-6-one was accomplished [26]. The first total synthesis of canthin-6-one (**1**), which was achieved with a poor overall yield via a classic Bischer-Napieralski method, was reported in 1966 [27]. In 2013, Hollis Showalter *et al.* reviewed the synthetic approaches to canthin-6-ones and their ring-truncated congeners [28], their review including almost all reported cases. It is noteworthy that these alkaloids have been shown to have broad potential biological activity, such as antitumor [9,11,13,29,30,31,32], antibacterial [33,34,35], antifungal [2,4,22,36,37,38], antiparasitic [16,39,40,41], antiviral [12,19,42,43,44], anti-inflammatory [10], antiproliferative [45,46], and aphrodisiac [47] properties, as well as uses in cancer chemoprevention [48], DNA screening [49], and reducing elevated levels of proinflammatory cytokines and nitric oxide production by lipopolysaccharide-stimulated macrophages [50]. Moreover, excellent photophysical properties were reported by Irikawa *et al.* in 1987 [51] and, more recently, by Taniguchi *et al.* in 2012 [15].

This review provides a broad overview of canthin-6-ones. The first section describes the biosynthetic line and the relevant biosynthetic assembly lines of canthin-6-one alkaloids, the second section considers classic and efficient synthetic methods, and the last section summarizes the excellent biological activity of canthin-6-one alkaloids.

## 2. Biosynthesis

### 2.1. Biosynthetic Pathway

**Scheme 1:**
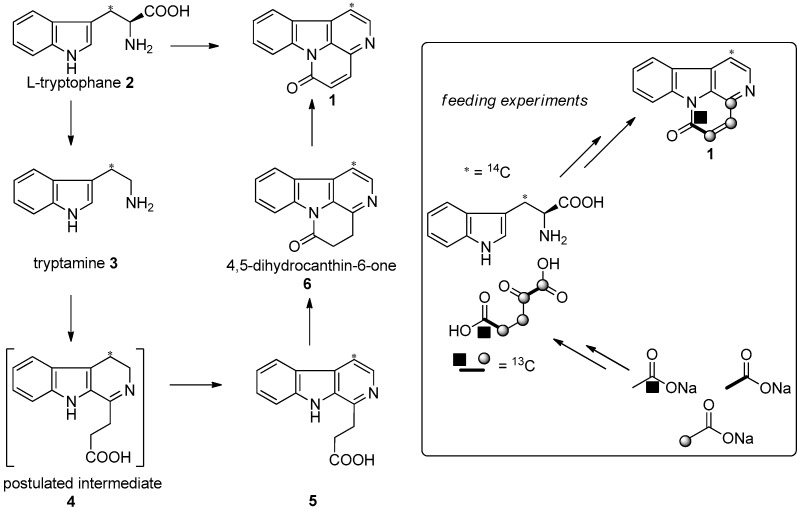
Biosynthetic mechanism leading to canthin-6-one alkaloids, and the supporting feeding experiments.

Guicciardi’s and Aragozzini’s groups were the first to demonstrate the biosynthesis of canthin-6-one alkaloids. A general biosynthetic pathway starting from tryptophan (**2**) is depicted in Scheme 1 [24,25]. All intermediates were characterized by the authors as products of the incorporation of [methylene-^14^C]-tryptophan (**2**). Dihydro-β-carboline-1-propionic acid (**4**) may be the first intermediate, with the decarboxylation of tryptophan (**2**) into tryptamine (**3**), and a suitable oxidation may generate β-carboline-1-propionic acid (**5**), which was isolated in the course of Guicciardi’s feeding experiments. The tricyclic intermediate (**5**) could be transformed into 4,5-dihydrocanthin-6-one (**6**), which may sequentially yield canthin-6-one (**1**) after oxidation. The pathway was confirmed by a supporting feeding experiment carried out in 1988 (inset in Scheme 1).

### 2.2. Biosynthetic Assembly Line

**Scheme 2:**
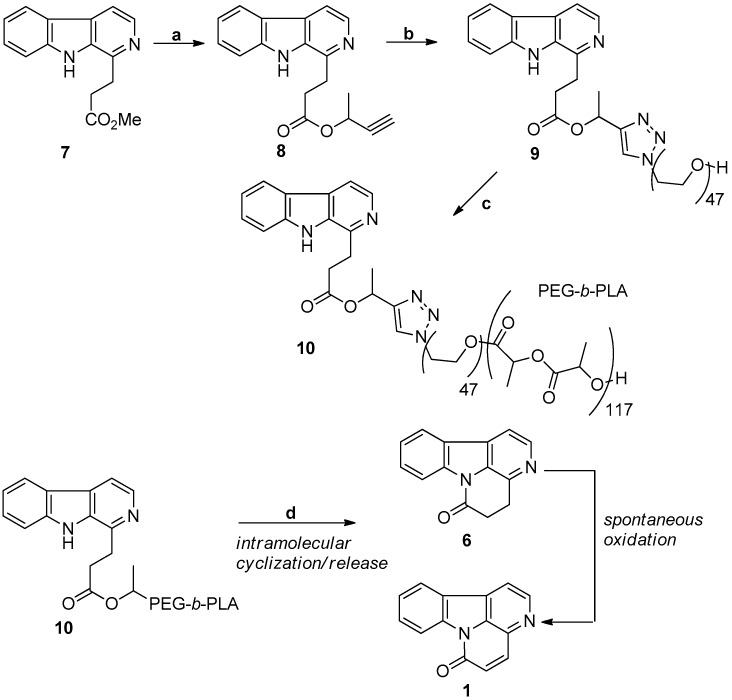
The relevant biosynthetic assembly lines. *Reagents and conditions*: **7**-functionalized nanoparticles and the biomimetic release of **1** and **6**. (**a**) DCM, Yb(Otf)_3_ (20 mol %), reflux, 16 h, 27%; (**b**) 3-butyn-2-ol (excess), N_3_-PEG-OH (1 equiv.), CuBr (1 equiv.), dimethylformamide, room temperature (RT), 24 h, PMDTA (*N*,*N*,*N*′,*N*″,*N*″-pentamethyldiethylenetriamine), 46%; (**c**) *d,l*-lactide (excess), Sn(Oct)_2_ (0.4 equiv.), toluene, 115 °C, 16 h, 57%; (**d**) 1,8-diazabicyclo[5.4.0]undec-7-ene (DBU) (excess), DCM, air, 72 h (identification of **1** and **6** by mass spectrometry and high-performance liquid chromatography-ultraviolet detection studies).

Based on the intimate biochemical mechanisms of the biosynthetic pathways, the first nanoparticle system to mimic the relevant biosynthetic assembly lines to canthin-6-one was elucidated by Cebrián-Torrejón *et al.* in 2013 (Scheme 2) [26]. The strategy used relied on: (i) the covalent linkage of infractine (**7**) by “click” chemistry to poly(ethylene glycol); (ii) the use of **7**-PEG-OH as a macroinitiator for the ring-opening polymerization of lactide to form **7**-PEG-b-PLA copolymer; (iii) the formation of **7**-PEG-b-PLA nanoparticles from the self-assembly of **7**-PEG-b-PLA in aqueous solution; and (iv) the potential biomimetic release of canthin-6-one (**1**) from the nanoparticles.

## 3. Chemistry

The canthin-6-one alkaloids have been synthesized via different approaches by many researchers [2,52,53,54,55,56,57,58,59,60,61,62,63,64,65,66,67,68,69,70,71,72,73,74,75]. In order to use these methods to guide our review more clearly, we categorized classic and efficient synthetic methods according to their key reaction steps.

### 3.1. Bischer-Napieralski Reaction

**Scheme 3:**
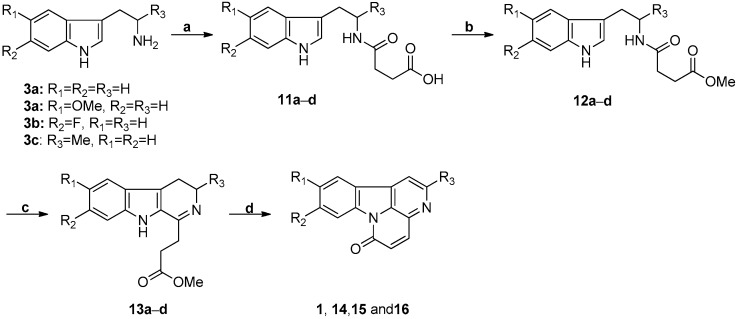
The synthetic strategy of Soriano-Agaton. *Reagents and conditions*. (**a**) Succinic anhydride, DCM, RT, 18 h, 98%, 99%, 98%, and 98% for **11a**−**d**, respectively; (**b**) Amberlyst 15, MeOH, reflux, 18 h, 98%; (**c**) POCl_3_, PhH, reflux, 1 h; (**d**) DBU, DCM, RT, 18 h, 80%, 70%, 80%, and 20% in two steps for **1**, **14**, **15**, and **16**, respectively.

The use of the Bischer-Napieralski reaction to synthesize canthin-6-one alkaloids was first reported in 1966 [27], and Soriano-Agaton *et al.* reported a more recent synthesis in 2005 (Scheme 3) [2]. The overall yield of canthin-6-one in the later synthesis was increased to 76.83% in four steps by Soriano-Agaton.

### 3.2. Pictet-Spengler Reaction

**Scheme 4:**
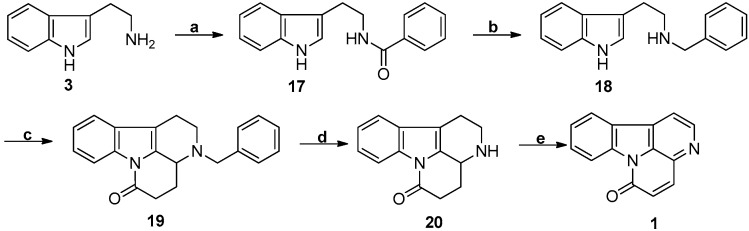
The synthetic strategy of Czerwinski. *Reagents and conditions.* (**a**) Anhydrous acetone, PhCOCl, NaOH, −30 °C, 95%; (**b**) anhydrous tetrahydrofuran (THF), LiAlH_4_, 0 °C—reflux, 95%; (**c**) α-ketoglutaric acid, PhH/dioxane (6:4), Dean-Stark trap, reflux, 80%; (**d**) anhydrous MeOH, Pd/C, HCOONH_4_, reflux, 83%; (**e**) PhH/PhMe (3:1), MnO_2_, reflux, 78%.

The Pictet-Spengler reaction was first used to synthesize canthin-6-one alkaloids by Mitscher *et al.* in 1975 [73], and many syntheses of canthin-6-one that employ this reaction have been reported since then. Czerwinski *et al.* [54] reported an efficient synthetic approach to canthin-6-one via the Pictet-Spengler reaction in 2003 (Scheme 4), obtaining an overall yield of 46.74%.

### 3.3. Diels-Alder Reaction

**Scheme 5:**
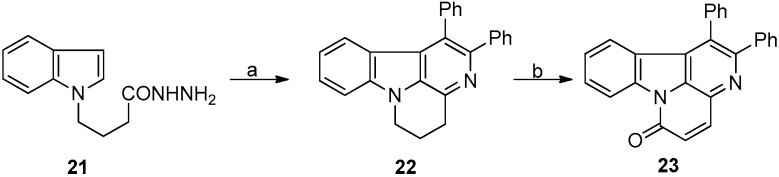
The synthetic strategy of Lindsley. *Reagents and conditions*. (**a**) 1,2-Diphenylethanedione, AcONH_4_, AcOH, 220 °C, 40 min, 80%; (**b**) 4,5-bis(thioacetamido)pentanoyl (BTAP), AcOH, 70 °C, 4 h, 60%.

In 1992, Snyder and co-workers reported an elegant strategy for accessing the canthine skeleton through using indole as a dienophile in an intramolecular inverse electron demand Diels-Alder (IEDDA) reaction [68]. Subsequently, in 2003, Lindsley *et al.* [70] reported a “one-pot” microwave-mediated synthesis of canthin-6-one analogs via the IEDDA reaction (Scheme 5), in which the overall yield was 48% in two steps.

### 3.4. Aldol Reaction

**Scheme 6:**
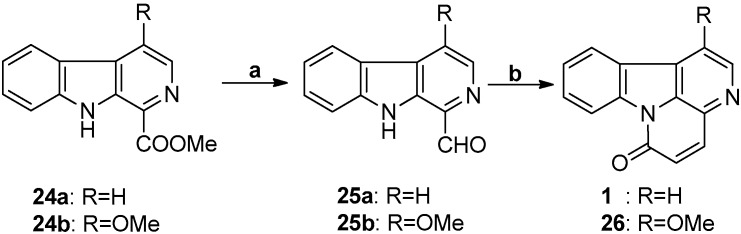
The synthetic strategy of Suzuki. *Reagents and conditions*. (**a**) Dibal-H, DCM, −40 °C, 5 min, 85% for **25a** and 70% for **25b**; (**b**) EtOAc/LiHMDS/THF, stirred for 15 min at −78 °C, then EtOH quench for 30 min at RT, 83% for **1** and 88% for **26**.

An efficient synthesis of canthin-6-one from β-carboline-1-carbaldehyde via the aldol reaction was reported by Suzuki *et al.* in 2005 (Scheme 6) [56]. Using a two-step reaction, canthin-6-one was obtained with an overall yield of 70.55%.

### 3.5. Perkin Reaction

**Scheme 7:**
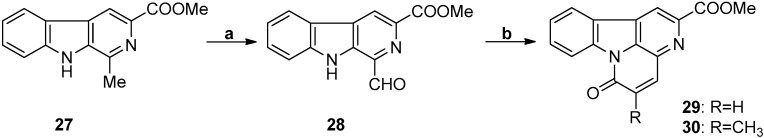
The synthetic strategy of Giudice. *Reagents and conditions*. (**a**) SeO_2_, dioxane, reflux, 2 h, 68%; (**b**) (RCO)_2_O, pyridine, heat, 3 h, 72% for **29** and 68% for **30**.

The use of the Perkin reaction to synthesize canthin-6-one alkaloid analogs was reported by Giudice *et al.* in 1990 (Scheme 7) [75] and, more recently, by Brahmbhatt *et al.* in 2010 [42]. Despite the relatively low overall yield (48.96% for **29** and 46.24% for **30**), the synthesis strategy is relatively simple and low-cost.

### 3.6. Non-Classic Strategy

**Scheme 8:**
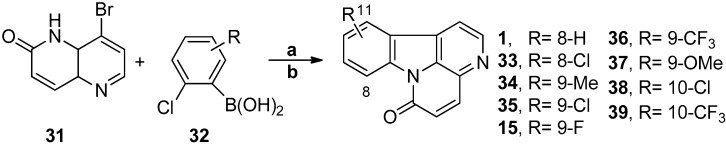
The synthetic strategy of Gollner. *Reagents and conditions*. (**a**) ArB(OH)_2_, K_2_CO_3_, Pd(dppf)Cl_2_, dioxane/H_2_O (3:1); (**b**) CuI, *N*,*N*′-dimethylethylendiamine (DMEDA), Cs_2_CO_3_, dioxane/H_2_O (3:1). Overall yields: **1**, 95%; **33**, 82%; **34**, 92%; **35**, 82%; **15**, 88%; **36**, 78%; **37**, 92%; **38**, 77%; **39**, 71%.

In 2010, Gollner *et al.* reported a “non-classic” strategy that focused on the construction of the central B ring (Scheme 8) [61]. The strategy relies on a palladium-catalyzed Suzuki-Miyaura C-C coupling followed by a copper-catalyzed C-N coupling that can be achieved either stepwise or in a new one-pot protocol starting from the appropriate 8-bromo-1,5-naphthyridine. Canthin-6-one (**1**) and nine analogues were prepared rapidly and in high yields (71%–95%). Ethyl canthin-6-one-1-carboxylate has also been efficiently synthesized, by Ioannidou *et al.* in 2011, from readily prepared ethyl 4-bromo-6-methoxy-1,5-naphthyridine-3-carboxylate in a three-step “non-classic” reaction that focuses on the construction of the central pyrrole (B ring) via a palladium-catalyzed Suzuki-Miyaura coupling followed by a copper-catalyzed C-N coupling; the overall yield was 85% [62].

## 4. Biological Activity

Canthin-6-one alkaloids have been reported to have a wide range of potential therapeutic applications, including, but not limited to, use as antitumor [9,11,13,29,30,31,32], antibacterial [33,34,35], antifungal [2,4,22,36,37,38], antiparasitic [16,39,40,41], antiviral [12,19,42,43,44], anti-inflammatory [10,76], antiproliferative [45,46], and aphrodisiac [47] agents, and use in cancer chemoprevention [48], DNA screening [49], reducing elevated levels of proinflammatory cytokines and nitric oxide production by lipopolysaccharide-stimulated macrophages [50], and so on. In this review, favorable biological activities of canthin-6-one alkaloids that are equal to or better than those of standard drugs are discussed, and their potential is highlighted.

### 4.1. Antibacterial

In 2007, O’Donnell *et al.* reported the *in vitro* antibacterial activity of canthin-6-one alkaloids, and found minimum inhibitory concentrations (MICs) in the range of 8–82 µg/mL against a panel of fast-growing *Mycobacterium* species and 8–64 µg/mL against multidrug-resistant and methicillin-resistant strains of *Staphylococcus aureus* [33]. In the same year, Ostrov *et al.* [34] reported the use of structure-based molecular docking to identify novel drug-like small molecules. They found that canthin-6-one could dock to two sites of *Escherichia coli* DNA gyrase, targeting and inhibiting the DNA supercoiling activity of purified *E. coli* DNA gyrase, although it could not effectively accumulate inside *E. coli*. Our group has designed and synthesized a series of 3-*N*-alkylated and 3-*N*-benzylated canthin-6-ones, and evaluated their *in vitro* antibacterial activities. Of these compounds, eleven 3-*N*-substituted canthin-6-ones were found to be the most potent, with MIC values lower than 1.95 (μg/mL) against *S. aureus* [77].

### 4.2. Antitumor

The significant antitumor activity of canthin-6-one alkaloids has been reported by many researchers. For example, in 2014, Devkota *et al.* [11] reported a study in which canthin-6-one alkaloids were tested in an *Nf1*- and *p53*-defective mouse malignant glioma tumor cell line engineered to express a dual reporter (Table 2). The results indicated that the majority of the canthin-6-one alkaloids inhibited cell growth and exhibited some toxicity. The antitumor mechanism was reported by Dejos * et al.* in the same year [45]. They found that the primary effect of canthin-6-one is as an antiproliferative, possibly by interfering with the G_2_/M transition. In 2015, Cebrian-Torrejon *et al.* [78] presented an approach for studying the performance of novel targets able to overcome cancer stem cell chemoresistance. The approach was based on voltammetric data for microparticulate films of natural or synthetic alkaloids from the canthin-6-one series.

### 4.3. Antifungal

**Figure 2:**
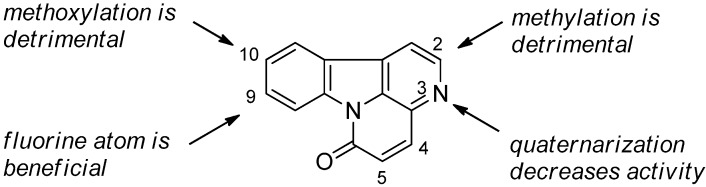
Structure-activity relationships for antifungal activity.

Canthin-6-one (**1**) was reported by Thouvenel et al. in 2003 to exhibit a broad spectrum of activity against *Aspergillus fumigatus*, *A. niger*, *A. terreus*, *Candida albicans*, *C. tropicalis*, *C. glabrata*, *C. neoformans*, *Geotrichum candidum*, *Saccharomyces cerevisiae*, *Trichosporon beigelii*, *T. cutaneum*, and *T. mentagrophytes* var. interdigitale, with MICs between 5.3 and 46 µM [36]. Moreover, in 2005, Soriano-Agaton *et al*. [2] described the structure -activity relationships for the antifungal activity of canthin-6-one (Figure 2). The mechanism of action of the antifungal canthin-6-one series was investigated in *Saccharomyces cerevisiae* by Loiseau *et al*. in 2008 [79]. In 2013, the antifungal mechanism of canthin-6-one was also reported on by Dejos *et al*. [38]. Although no novel clues to the mechanism were found, they demonstrated that the major-facilitator-superfamily (MFS)-type transporter Flr1 may be able to reduce sensitivity to canthin-6-one when it is overproduced via a higher gene dosage, and that Flr1-mediated tolerance to canthin-6-one is strictly dependent on the transcription factor Yap1. As Dejos *et al*. stated, “Although the Yap1-Flr1 pair is not naturally involved in yeast tolerance to canthin-6-one, this study demonstrates that their overexpression can lead to resistance to the chemical stress generated by this drug.”

### 4.4. Anti-Inflammatory

The transcription factor NF-κB is a key regulator of many proinflammatory pathways, and therefore its inhibition results in anti-inflammatory effects. Canthin-6-one alkaloids were first found to be NF-κB inhibitors by Tran *et al.* in 2014 [10]. The IC_50_ values of 9-hydroxycanthin-6-one and 9-methoxycanthin-6-one were 3.8 and 7.4 µM, respectively. However, the IC_50_ value of the standard drug parthenolide was only 1.5 µM.

### 4.5. Wnt Signaling Inhibitors

Numerous diseases have been attributed to the aberrant transduction of Wnt signaling, which regulates various processes such as cell proliferation and differentiation, and embryo development. In 2015, 9-hydroxycanthin-6-one was screened for its activity in targeting TCF/β-catenin transcriptional modulating activity with a cell-based luciferase assay by Ohishi *et al.* [80]. The degradation of β-catenin by 9-hydroxycanthin-6-one was suppressed by GSK3β-siRNA, while 9-hydroxycanthin-6-one decreased β-catenin even in the presence of CK1*α*-siRNA. These results suggest that 9-hydroxycanthin-6-one inhibits Wnt signaling through the activation of GSK3β, independently of CK1*α*.

### 4.6. Protein Tyrosine Phosphatase 1B Inhibitors

**Figure 3:**
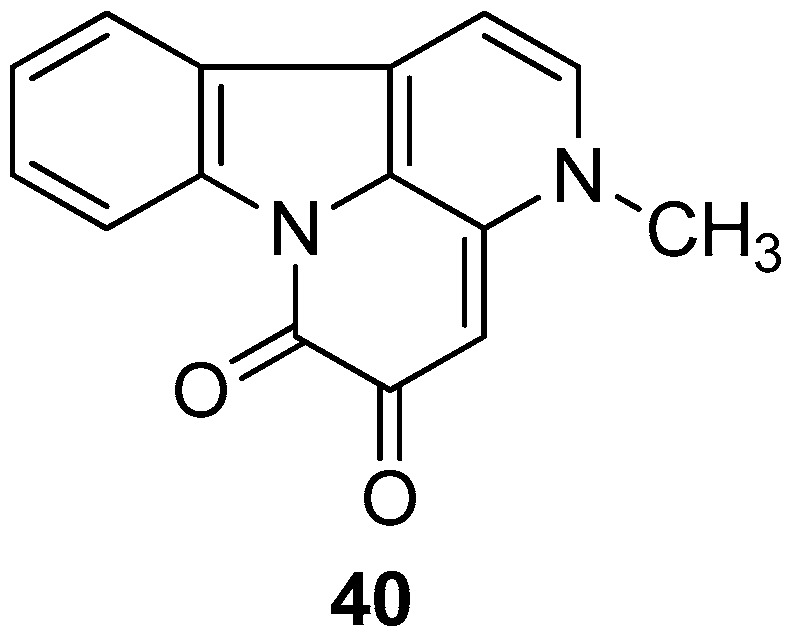
Structure of canthine **40**.

As a potential therapy for diabetes, protein tyrosine phosphatase 1B (PTP1B) inhibitors have attracted considerable attention. In 2015, Sasaki *et al.* [81] reported that compound **40** (Figure 3) is the competitive PTP1B inhibitor, with the best inhibitory selectivity of PTP1B and other protein tyrosine phosphatase (PTPs), and showed in cell-based assays that it promotes activity in the insulin signaling pathway.

## 5. Conclusions and Future Prospects

The main achievements in the study of canthin-6-one alkaloids over the period of 1952 to 2015 have been reviewed, with emphasis on the biosynthesis, chemistry, and biological activities of these compounds. The low toxicity and good biological activities of canthin-6-one alkaloids mean that there is potential for them to be developed into new drugs. New research by Doménech-Carbó *et al.* has shown that microparticulate films of canthin-6-one on glassy carbon electrodes could yield separate voltammetric signals for dsDNA, ssDNA, and G-quadruplex DNA, different degrees of DNA methylation, and biomimetic nucleosomal DNA, with a detection limit of 10^−5^ M [49]. Moreover, excellent photophysical properties of canthin-6-one have been recently reported by Taniguchi *et al.* [15].

Further research by international groups is required to: (i) develop efficient and environmentally sustainable synthetic strategies for producing canthin-6-one alkaloids on a large scale; (b) elucidate the structure-activity relationships and mechanisms of the different biological activities of these compounds; (c) explore their biological activities; and (d) identify electrochemical and photochemical applications.

## Figures and Tables

**Table 1:** Representative canthin-6-one alkaloids isolated from natural sources.

When Isolated	Natural Canthin-6-one Alkaloids
1952–1999 (**25**)	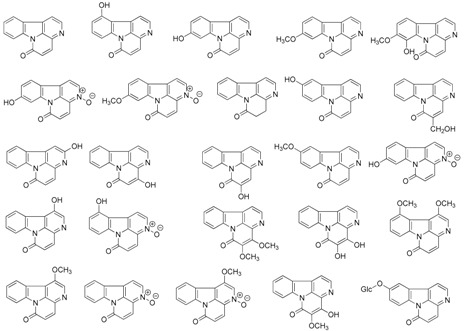
2000–2004 (**16**)	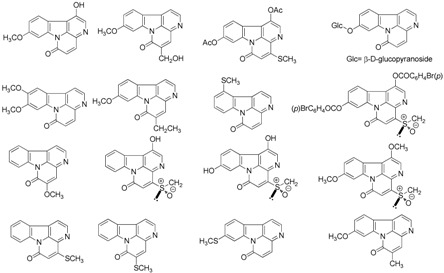
2005–2015 (**19**)	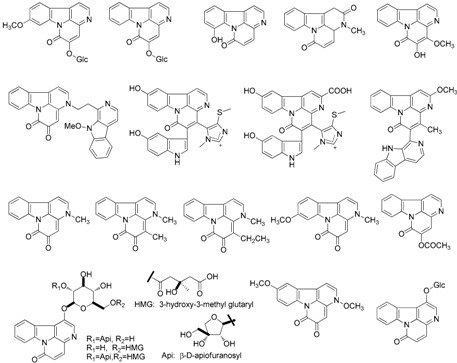

**Table 2:** Cell viability and inhibition of proliferation (%) in an *Nf1*- and *p53*-defective mouse Central Nervous System (CNS) tumor cell line by canthines at 2.0 mg/mL.

Compound	Cell Viability (Untreated Controls = 100)	Inhibition of Proliferation (%)
Canthin-6-one-9-methoxy-5-*O*-β-d-glucopyranoside	51	71
9-Methoxycanthin-6-one	41	76
8-Hydroxy-9-methoxycanthin-6-one	51	59
9-Hydroxycanthin-6-one	56	48
canthin-6-one-3-*N*-oxide	59	70
9-Hydroxycanthin-6-one-3-*N*-oxide	54	76
11-Hydroxycanthin-6-one-3-*N*-oxide	54	70

## References

[1] Tessier A., Campbell P.G., Bisson M. (1979). Sequential extraction procedure for the speciation of particulate trace metals. Anal. Chem..

[2] Soriano-Agatón F., Lagoutte D., Poupon E., Roblot F., Fournet A., Gantier J.C., Hocquemiller R. (2005). Extraction, hemisynthesis, and synthesis of canthin-6-one analogues. Evaluation of their antifungal activities. J. Nat. Prod..

[3] Mukhlesur R.M., Anwarul I.M., Khondkar P., Gray A.I. (2005). Alkaloids and lignans from *Zanthoxylum** budrunga* (Rutaceae). Biochem. Syst. Ecol..

[4] He W., Puyvelde L.V., Kimpe N.D., Verbruggen L., Anthonissen K., Flaas M.V., Bosselaers J., Mathenge S.G., Mudida F.P. (2002). Chemical constituents and biological activities of *Zanthoxylum** usambarense*. Phytother. Res..

[5] Cebrián-Torrejón G., Kablan L., Ferreira M.E., Cruz D.R., Doménech-Carbó A., Bilbao N.V., Arias A.R., Figadère B., Poupon E., Fournet A. (2015). Harvesting canthinones: Identification of the optimal seasonal point of harvest of *Zanthoxylum chiloperone* leaves as a source of 5-methoxycanthin-6-one. Nat. Prod. Res..

[6] Min Y.D., Kwon H.C., Yang M.C., Lee K.H., Choi S.U., Lee K.R. (2007). Isolation of limonoids and alkaloids from Phellodendron amurense and their multidrug resistance (MDR) reversal activity. Arch. Pharm. Res..

[7] Shi M.J., Liu W.W., Wang H.Y., Cheng H., Hu L. (2000). Effect of canthin-6-one experimental arrhythmia. Chin. Tradit. Herba. Drugs.

[8] Ferreira M.E., Cebrián-Torrejón G., Corrales A.S., de Bilbao N.V., Rolón M., Gomez C.V., Leblanc K., Yaluf G., Schinini A., Torres S. (2011). *Zanthoxylum*
*chiloperone* leaves extract: First sustainable Chagas disease treatment. J. Ethnopharmacol..

[9] Rivero-Cruz J.F., Lezutekong R., Lobo-Echeverri T., Ito A., Mi Q., Chai H.B., Soejarto D.D., Cordell G.A., Pezzuto J.M., Swanson S.M. (2005). Cytotoxic constituents of the twigs of *Simarouba glauca* collected from a plot in southern florida. Phytother. Res..

[10] Tran T.V.A., Malainer C., Schwaiger S., Atanasov A.G., Heiss E.H., Dirsch V.M., Stuppner H. (2014). NF-κB inhibitors from *Eurycoma** longifolia*. J. Nat. Prod..

[11] Devkota K.P., Wilson J.A., Henrich C.J., McMahon J.B., Reilly K.M., Beutler J.A. (2014). Compounds from *Simarouba** berteroana* which inhibit proliferation of NF1-defective cancer cells. Phytochem. Lett..

[12] Chen J., Yan X.H., Dong J.H., Sang P., Fang X., Di Y.T., Zhang Z.K., Hao X.J. (2009). Tobacco mosaic virus (TMV) inhibitors from *Picrasma** quassioides* Benn. J. Agric. Food Chem..

[13] Kuo P.C., Shi L.S., Damu A.G., Su C.R., Huang C.H., Ke C.H., Wu .B., Lin A.J., Bastow K.F., Lee K.H. (2003). Cytotoxic and antimalarial β-carboline alkaloids from the roots of *Eurycoma** longifolia*. J. Nat. Prod..

[14] Handa S.S., Kinghorn A.D., Cordell G.A., Farnsworth N.R. (1983). Plant anticancer agents XXV. Constituents of *Soulamea** soulameoides*. J. Nat. Prod..

[15] Taniguchi K., Takizawa S., Hirano T., Murata S., Kagechika H., Kishida A., Ohsaki A. (2012). Amarastelline A: A fluorescent alkaloid from *Quassia** amara* and its properties in living cells. ChemPlusChem.

[16] Kardono L.B., Angerhofer C.K., Tsaur S., Padmawinata K., Pezzuto J.M., Kinghorn A.D. (1991). Cytotoxic and antimalarial constituents of the roots of *Eurycoma** longifolia*. J. Nat. Prod..

[17] Kanchanapoom T., Kasai R., Chumsri P., Hiraga Y., Yamasaki K. (2001). Canthin-6-one and β-carboline alkaloids from *Eurycoma** harmandiana*. Phytochemistry.

[18] Bharitkar Y.P., Hazra A., Apoorva Poduri N.S., Ash A., Maulik P.R., Mondal N.B. (2015). Isolation, structural elucidation and cytotoxicity evaluation of a new pentahydroxy-pimarane diterpenoid along with other chemical constituents from *Aerva lanata*. Nat. Prod. Res..

[19] Hsieh P.W., Chang F.R., Lee K.H., Hwang T.L., Chang S.M., Wu Y.C. (2004). A new anti-HIV alkaloid, drymaritin, and a new c-glycoside flavonoid, diandraflavone, from *Drymaria diandra*. J. Nat. Prod..

[20] Ma Z.Z., Hano Y., Nomura T., Chen Y.J. (2000). Alkaloids and phenylpropanoids from *Peganum** nigellastrum*. Phytochemistry.

[21] Bröckelmann M.G., Dasenbrock J., Steffan B., Steglich W., Wang Y., Raabe G., Fleischhauer J. (2004). An unusual series of thiomethylated canthin-6-ones from the North American mushroom *Boletus curtisii*. Eur. J. Org. Chem..

[22] Tanaka N., Momose R., Takahashi Y., Kubota T., Takahashi-Nakaguchi A., Gonoi T., Fromont J., Kobayashi J. (2013). Hyrtimomines D and E, bisindole alkaloids from a marine sponge *Hyrtios* sp.. Tetrahedron Lett..

[23] Haynes H.F., Nelson E.R., Price J.R. (1952). Alkaloids of the Australian Rutaceae: *Pentaceras australis* Hook. F.I. Isolation of the Alkaloids and Identification of Canthin-6-one. Aust. J. Sci. Res. Ser. A.

[24] Crespi-Perellino N., Guicciardi A., Malyszko G., Minghetti A. (1986). Biosynthetic relationship between indole alkaloids produced by cell cultures of *Ailanthus altissima*. J. Nat. Prod..

[25] Aragozzini F., Maconi E., Gualandris R. (1988). Evidence for involvement of ketoglutarate in the biosynthesis of canthin-6-one from cell cultures of *Ailanthus altissima*. Plant Cell Rep..

[26] Cebrián-Torrejón G., Mackiewicz N., Vázquez-Manrique R.P., Fournet A., Figadère B., Nicolas J., Poupon E. (2013). Solution phase and nanoparticular biosynthetically inspired interconnections in the canthi-6-one β-carboline series and study of phenotypic properties on *C. elegans*. Eur. J. Org. Chem..

[27] Rosenkranz H.J., Botyos G., Schmid H. (1966). Synthese von tuboflavin, 4-Äthyl-canthin-6-one and canthin-6-one. Justus Liebigs Ann. Chem..

[28] Hollis Showalter H.D. (2013). Progress in the synthesis of canthine alkaloids and ring-truncated congeners. J. Nat. Prod..

[29] Peduto A., More V., de Caprariis P., Festa M., Capasso A., Piacente S., de Martino L., de Feo V., Filosa R. (2011). Synthesis and cytotoxic activity of new β-carboline derivatives. Mini-Rev. Med. Chem..

[30] Miyake K., Tezuka Y., Awale S., Li F., Kadota S. (2010). Canthin-6-one alkaloids and a tirucallanoid from *Eurycoma*
*longifolia* and their cytotoxic activity against a human HT-1080 fibrosarcoma cell line. Nat. Prod. Commun..

[31] Ammirante M., di Giacomo R., de Martino L., Rosati A., Festa M., Gentilella A., Pascale M.C., Belisario M.A., Leone A., Turco M.C. (2006). 1-Methoxy-canthin-6-one induces c-Jun NH_2_-terminal kinase-dependent apoptosis and synergizes with tumor necrosis factor-related apoptosis-inducing ligand activity in human neoplastic cells of hematopoietic or endodermal origin. Cancer Res..

[32] Kuo P.C., Damu A.G., Lee K.H., Wu T.S. (2004). Cytotoxic and antimalarial constituents from the roots of *Eurycoma** longifolia*. Bioorg. Med. Chem..

[33] O’Donnell G., Gibbons S. (2007). Antibacterial activity of two canthin-6-one alkaloids from *Allium neapolitanum*. Phytother. Res..

[34] Ostrov D.A., Prada J.A.H., Corsino P.E., Finton K.A., Le N., Rowe T.C. (2007). Discovery of novel DNA gyrase inhibitors by high-throughput virtual screening. Antimicrob. Agents Chemother..

[35] Chen M., Fan H.Y., Dai S.J., Liu K. (2007). Alkaloids from twigs and leaves of *Picrasma** quassioides*. Chin. Tradit. Herbal Drugs.

[36] Thouvenel C., Gantier J.C., Duret P., Fourneau C., Hocquemiller R., Ferreira M.E., de Arias A.R., Fournet A. (2003). Antifungal compounds from *Zanthoxylum** chiloperone* var. *angustifolium*. Phytother. Res..

[37] Li H.Y., Koike K., Ohmoto T. (1993). New alkaloids, picrasidines W, X and Y, from *Picrasma** quassioides* and X-ray crystallographic analysis of picrasidine Q. Chem. Pharm. Bull..

[38] Dejos C., Régnacq M., Bernard M., Voisin P., Bergès T. (2013). The MFS-type efflux pump Flr1 induced by Yap1 promotes canthin-6-one resistance in yeast. Fed. Eur. Biochem. Soc. Lett..

[39] Ferreira M.E., Nakayama H., de Arias A.R., Schinini A., de Bilbao N.V., Serna E., Lagoutte D., Soriano-Agatón F., Poupon E., Hocquemiller R. (2007). Effects of canthin-6-one alkaloids from *Zanthoxylum** chiloperone* on trypanosoma cruzi-infected mice. J. Ethnopharmacol..

[40] Takasu K., Shimogama T., Saiin C., Kim H.S., Wataya Y., Brun R., Ihara M. (2005). Synthesis and evaluation of β-carbolinium cations as new antimalarial agents based on π-delocalized lipophilic cation (DLC) hypothesis. Chem. Pharm. Bull..

[41] Ferreira M.E., de Arias A.R., de Ortiz S.T., Inchausti A., Nakayama H., Thouvenel C., Hocquemiller R., Fournet A. (2002). Leishmanicidal activity of two canthin-6-one alkaloids, two major constituents of *Zanthoxylum chiloperone* var. *angustifolium*. J. Ethnopharmacol..

[42] Brahmbhatt K.G., Ahmed N., Sabde S., Mitra D., Singh I.P., Bhutani K.K. (2010). Synthesis and evaluation of β-carboline derivatives as inhibitors of human immunodeficiency virus. Bioorg. Med. Chem. Lett..

[43] Xu Z., Chang F.R., Wang H.K., Kashiwada Y., McPhail A.T., Bastow K.F., Tachibana Y., Cosentino M., Lee K.H. (2000). Anti-HIV agents 451 and antitumor agents 205.2 two new sesquiterpenes, *Leitneridanins* A and B, and the cytotoxic and anti-HIV principles from *Leitneria floridana*. J. Nat. Prod..

[44] Ohomoto T., Koike K. (1984). Studies on the constituents of *Ailanthus altissima Swingle*. III. The alkaloidal constituents. Chem. Pharm. Bull..

[45] Dejos C., Voisin P., Bernard M., Régnacq M., Bergès T. (2014). Canthin-6-one displays antiproliferative activity and causes accumulation of cancer cells in the G2/M phase. J. Nat. Prod..

[46] De Feo V., Martino L.D., Santoro A., Leone A., Pizza C., Franceschelli S., Pascale M. (2005). Antiproliferative effects of tree of heaven (*Ailanthus altissima Swingle*). Phytother. Res..

[47] Chiou W.F., Wu T.S. (2012). 9-Hydroxycanthin-6-one induces penile erection and delays ejaculation. J. Sex. Med..

[48] Murakami C., Fukamiya N., Tamura S., Okano M., Bastow K.F., Tokuda H., Mukainaka T., Nishino H., Lee K.H. (2004). Multidrug-resistant cancer cell susceptibility to cytotoxic quassinoids, and cancer chemopreventive effects of quassinoids and canthine alkaloids. Bioorg. Med. Chem..

[49] Doménech-Carbó A., Cebrián-Torrejón G., de Miguel L., Tordera V., Rodrigues-Furtado D., Assad-Kahn S., Fournet A., Figadère B., Vázquez-Manrique R.P., Poupon E. (2014). DsDNA, ssDNA, G-quadruplex DNA, and nucleosomal DNA electrochemical screening using canthin-6-one alkaloid-modified electrodes. Electrochim. Acta.

[50] Siveen K., Kuttan G. (2012). Modulation of humoral immune responses and inhibition of proinflammatory cytokines and nitric oxide production by 10-methoxycanthin-6-one. Immunopharmacol. Immunotoxicol..

[51] Irikawa H., Okumura Y. (1987). Autoxidation of indolo [3,2,1-*de*][1,5]naphthyridines. Bull. Chem. Soc. Jpn..

[52] Hagen T.J., Cook J.M. (1988). Synthesis of 1-methoxycanthine-6-one. Tetrahedron Lett..

[53] Hagen T.J., Narayanan K., Names J., Cook J.M. (1989). DDQ oxidations in the indole area. Synthesis of 4-alkoxy-β-carbolines including the natural products crenatine and 1-methoxycanthin-6-one. J. Org. Chem..

[54] Czerwinski K.M., Zificsak C.A., Stevens J., Oberbeck M., Randlett C., King M., Mennen S. (2003). An improved synthesis of canthin-6-one. Synth. Commun..

[55] Rößler U., Blechert S., Steckhan E. (1999). Single electron transfer induced total synthesis of canthin-6-one. Tetrahedron Lett..

[56] Suzuki H., Adachi M., Ebihara Y., Gyoutoku H., Furuya H., Murakami Y., Okuno H. (2005). A total synthesis of 1-methoxycanthin-6-one: An efficient one-pot synthesis of the canthin-6-one skeleton from β-Carboline-1-carbaldehyde. Synthesis.

[57] Markgraf J.H., Dowst A.A., Hensley L.A., Jakobsche C.E., Kaltner C.J., We P.J., Zimmerman P.W. (2005). A versatile route to benzocanthinones. Tetrahedron.

[58] Bergman J., Lidgren G., Gogoll A. (1992). Synthesis and reactions of oxazolones from l-tryptophan and α-haloacetic anhydrides. Bull. Soc. Chim. Belg..

[59] Condie G.C., Bergman J. (2004). Reactivity of β-carbolines and cyclopenta[*b*]indolones prepared from the intramolecular cyclization of 5(4*H*)-oxazolones derived from l-tryptophan. Eur. J. Org. Chem..

[60] Singh V., Hutait S., Batra S. (2009). Baylis-Hillman reaction of 1-formyl-β-carboline: One-step synthesis of the canthin-6-one framework by an unprecedented cascade cyclization reaction. Eur. J. Org. Chem..

[61] Gollner A., Koutentis P.A. (2010). Two-step total syntheses of canthin-6-one alkaloids: New one-pot sequential Pd-catalyzed Suzuki-Miyaura coupling and Cu-catalyzed amidation reaction. Org. Lett..

[62] Ioannidou H.A., Martin A., Gollner A., Koutentis P.A. (2011). Three-step synthesis of ethyl canthinone-3-carboxylates from ethyl 4-bromo-6-methoxy-1,5-naphthyridine-3-carboxylate via a Pd-catalyzed Suzuki-Miyaura coupling and a Cu-catalyzed amidation reaction. J. Org. Chem..

[63] Markgraf J.H., Snyder S.A., Vosburg D.A. (1998). A concise route to isocanthin-6-one. Tetrahedron Lett..

[64] Snyder S.A., Vosburg D.A., Jarvis M.G., Markgraf J.H. (2000). Intramolecular hetero Diels-Alder routes to γ-carboline alkaloids. Tetrahedron.

[65] Hájíček J., Trojánek J. (1982). Synthesis of optically active 4,4- and 5,5-disubstituted 4,5-dihydro-6*H*-canthin-6-ones and their CD spectra. Collect. Czechoslov. Chem. Commun..

[66] Hájíček J., Holubek J., Trojánek J. (1982). Synthesis of 4,4- and 5,5-disubstituted 4,5-dihydro-6H-canthin-6-ones. Collect. Czechoslov. Chem. Commun..

[67] Chughtai M., Eagan J.M., Padwa A. (2011). Intramolecular [4 + 2]-cycloaddition of 5-amino-substituted oxazoles as an approach toward the left-hand segment of haplophytine. Synlett.

[68] Benson S.C., Li J.H., Snyder J.K. (1992). Indole as a dienophile in inverse electron demand Diels-Alder reactions. intramolecular reactions with 1,2,4-triazines to access the canthine skeleton. J. Org. Chem..

[69] Li J.H., Snyder J.K. (1994). Selective oxidation of canthines to canthin-6-ones with triethylbenzylammonium permanganate. Tetrahedron Lett..

[70] Lindsley C.W., Wisnoski D.D., Wang Y., Leister W.H., Zhao Z. (2003). A “one pot” microwave-mediated synthesis of the basic canthine skeleton: Expedient access to unnatural β-carboline alkaloids. Tetrahedron Lett..

[71] De Bruyn A., Eeckhaut G., Villaneuva J., Hannart J. (1985). Synthesis and ^1^H-NMR study of 3,4-diethyl-1,2,3,3a,4,5-hexahydro-canthinone-6. Tetrahedron.

[72] Puzik A., Bracher F. (2009). A convenient approach to the canthin-4-one ring system: Total synthesis of the alkaloids tuboflavine and norisotuboflavine. J. Heterocycl. Chem..

[73] Mitscher L., Shipchandler M., Showaleter H., Bathala M. (1975). Antimicrobial agents from higher-plants-synthesis in canthin-6-one (6*H*-indolo[3,2,1-de][1,5]naphthyridin-6-one) series. Heterocycles.

[74] Fang H.W., Liao Y.R., Hwang T.L., Shieh P.C., Lee K.H., Hung H.Y., Wu T.S. (2015). Total synthesis of cordatanine, structural reassignment of drymaritin, and anti-inflammatory activity of synthetic precursors. Bioorg. Med. Chem. Lett..

[75] Giudice M.R.D., Settimj F.G.G. (1990). New tetracyclic compounds containing the β-carboline moiety. J. Heterocycl. Chem..

[76] Kim H.M., Kim S.J., Kim H.Y., Ryu B., Kwak H., Hur J., Choi J.H., Jang D.S. (2015). Constituents of the stem barks of Ailanthus altissima and their potential to inhibit LPS-induced nitric oxide production. Bioorg. Med. Chem. Lett..

[77] Dai J.K., Dan W.J., Li N., Du H.T., Zhang J.W., Wang J.R. (2016). Synthesis, *in vitro* antibacterial activities of a series of 3-*N*-substituted canthin-6-ones. Bioorg. Med. Chem. Lett..

[78] Cebrian-Torrejon G., Domenech-Carbo A., Scotti M.T., Fournet A., Figadere B., Poupon E. (2015). Experimental and theoretical study of possible correlation between the electrochemistry of canthin-6-one and the anti-proliferative activity against human cancer stem cells. J. Mol. Struct..

[79] Lagoutte D., Nicolas V., Poupon E., Fournet A., Hocquemiller R., Libong D., Chaminade P., Loiseau P.M. (2008). Antifungal canthin-6-one series accumulate in lipid droplets and affect fatty acid metabolism in *Saccharomyces cerevisiae*. Biomed. Pharmacother..

[80] Ohishi K., Toume K., Arai M.A., Koyano T., Kowithayakorn T., Mizoguchi T., Itoh M., Ishibashi M. (2015). 9-Hydroxycanthin-6-one, a β-Carboline Alkaloid from *Eurycoma*
*longifolia*, Is the First Wnt Signal Inhibitor through Activation of Glycogen Synthase Kinase 3β without Depending on Casein Kinase 1α. J. Nat. Prod..

[81] Sasaki T., Li W., Higai K., Koike K. (2015). Canthinone alkaloids are novel protein tyrosine phosphatase 1B inhibitors. Bioorg. Med. Chem. Lett..

